# Enhanced Acceptance Specification of Asphalt Binder to Drive Sustainability in the Paving Industry

**DOI:** 10.3390/ma14226828

**Published:** 2021-11-12

**Authors:** Yiming Li, Simon A. M. Hesp

**Affiliations:** 1 Department of Civil Engineering, Northeast Forestry University, Harbin 150040, China; 2 Department of Chemistry, Queen’s University, Kingston, ON K7L 3N6, Canada; simon@chem.queensu.ca

**Keywords:** asphalt performance grading, thermal cracking, fatigue, phase angle, creep rate, failure strain

## Abstract

Testing small amounts of extracted and recovered asphalt binder as used in construction allows for the acceptance of materials in accordance with traffic and climate requirements. This approach facilitates the sustainable use of resources and thus prepares the paving industry for the true circular economy. Oscillatory, creep, and failure tests in a rheometer are compared for the performance grading of 32 asphalt binders extracted and recovered from real-world contract samples. Films 8 mm in diameter and 0.5 mm thick were tested from 35 to −5 °C in dynamic shear, followed by shear creep at 0 and 5 °C, and finally in tertiary tensile creep at 15 °C. The enhanced protocol uses a very small amount of material in contrast to current methods, yet it provides comparable results. Phase angle measurements appear to be optimal for performance grading, but further field study is required to determine if additional binder properties such as stiffness and/or failure strain would be required for the control of cracking.

## 1. Introduction

Optimal pavement design involves balancing material properties and structure to provide a long-life cycle with only minimal distress. It is generally accepted that rutting and moisture damage are largely controlled through the selection of appropriate aggregate types and gradation, with the addition of polymer, fiber, and/or antistrip additives when needed [1]. On the other hand, load-induced fatigue and cold temperature transverse cracking are kept in check by the selection of an appropriate pavement thickness, asphalt binder quality, and durability [1,2,3,4,5,6].

It is essential that the most accurate acceptance specification tests are conducted on carefully extracted and recovered binder, as it best reflects what is actually placed in the contract [7,8,9,10]. The presence of reclaimed asphalt pavement (RAP) in the mix, as well as associated overheating of the virgin asphalt binder during production, are factors that can have a detrimental impact on long-term performance of the pavement. Hence, these and other issues need to be accounted for in an effective quality assurance testing program.

**Figure 1:**
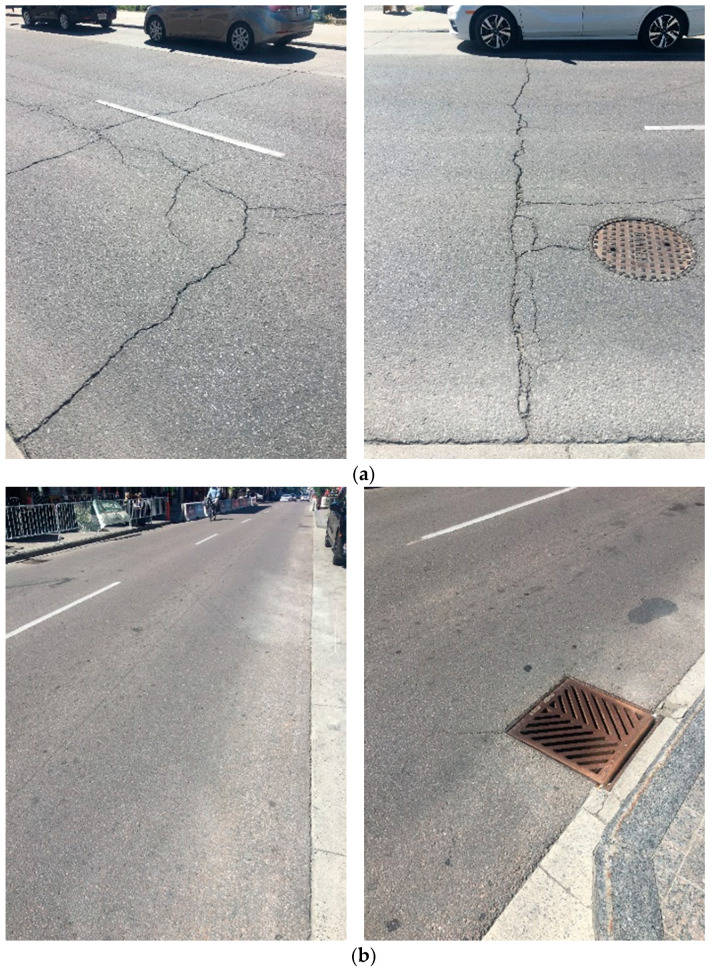
(**a**) Representative photographs of pavement reconstructed in 2010 on three blocks of Princess Street and King Street in downtown Kingston, Ontario, (**b**) Representative photographs of pavement reconstructed in 2011–2012 on seven adjacent blocks of Princess Street.

Several Ontario municipalities have recently switched to testing of the extracted and recovered asphalt binder according to the extended bending beam rheometer (EBBR) and double-edge-notched tension (DENT) tests with promising results [7,8,9,10]. To illustrate, Figure 1a provides representative 2020 photographs for pavement on three blocks of Princess Street and King Street in downtown Kingston, Ontario, constructed before the switch. The asphalt surface was reconstructed on fresh granular base ten years ago as part of a program that replaced all downtown sewer infrastructure. The properties of the asphalt storage tank sample were used for acceptance, but—according to the current Ontario municipal asphalt specification—up to 15% RAP was allowed in the binder course and none in the surface. It is obvious that this pavement is failing through thermal cracking well before its expected design life.

Figure 1b provides representative photographs for the remainder of the contract completed over the next two years on an adjacent seven blocks of Princess Street. Here, the City of Kingston had switched to acceptance of the asphalt based on properties of the extracted and recovered binder and RAP was banned from both the surface and binder courses. It is obvious that there is a stark contrast in performance after only eight to ten years of service. Hence, if the acceptance is based on extracted and recovered binder properties, then the use of RAP would be allowed as long as minimum performance is obtained in the materials as placed in the contract.

The acceptance of the asphalt for the City of Kingston has been based on extracted and recovered binder properties since 2010 and this has so far provided pavements that are meeting their design expectations [7]. While providing improved specification grading, the EBBR and DENT tests used for contract acceptance have their drawbacks. Both tests require a rather large quantity of extracted and recovered binder and take a considerable amount of time to complete. Hence, current research is focused on the development of simplified methods that use less material, take less time to complete, and with equal or better precision and accuracy [7,11,12,13]. Improved specification tests lower risk, which benefits both users and producers of asphalt for the betterment of the entire industry. The objective of the current research project is to develop a more practical approach for acceptance testing of extracted and recovered asphalt binder. In order to learn more about all issues involved, the research assessed a wide range of performance properties for 32 binders from commercial contracts. Future efforts will involve a detailed performance assessment of the involved pavement locations.

## 2. Background

It was Dow [14] of the Washington, D.C., engineering department, who in the early 1900s developed both a ductility test and an improved penetrometer for the grading of asphalt binder. With a keen eye, he had noticed that those binders that elongate when pulled by hand would perform better than those that ruptured early [15]. Both ductility and penetration tests went on to become the most widely used specification methods for straight asphalt binder and remain used with great success in many parts of the world today. In general, binder that flows well suffers little from cracking at ambient and cold temperatures. Dow [14] also commented on the fact that some binders when freshly poured perform much better compared to those that had been left to equilibrate for a time on the bench.

It was not until the publication from Hubbard and Pritchard [16] in 1916 that a quantitative assessment was made of this gradual aging phenomenon. Penetration testing showed that the consistency of binders can increase (i.e., penetration decreases) for periods of weeks and months, and that this was independent of oxidative hardening as reheating the sample could largely restore the original properties.

A series of publications by Traxler and coworkers [17,18,19] in the 1930s were the first to provide a comprehensive assessment of what is best described as a thermoreversible aging effect. Using tensile and shear creep experiments, major changes in rheological properties were revealed after days and weeks of isothermal storage. These authors noted that binders physically age at different rates depending on their source and production technology. They found that air oxidized binders were particularly sensitive to the effects of thermal conditioning. Filler had little effect on the degree of aging. Reheating could erase the changes. The impact of thermal equilibration was large compared to the effects of volatilization and oxidation. Finally, they described the change in consistency as a sol-to-gel transition.

Traxler and his contemporaries actively discussed the sol and gel nature of asphalt binder, as reflected by publications of Nellensteyn [20,21,22], Sakhanov [23], Sachanen [24], Mack [25,26], Saal [27], Pfeiffer and Van Doormaal [28], and many others. Asphalt binder is a material that is composed of a spectrum of organic molecules with molar weights that range from a few hundred to a few thousand grams per mole [29]. The individual molecules can be classified as either aliphatic (paraffins and naphthenes) or combined aliphatic and aromatic (naphthene aromatics, resins, and asphaltenes). The aliphatic fraction is defined by its molar weight and degree of branching, with those binders largely composed of linear alkanes (paraffins) providing lesser performance compared to those containing mostly branched and cyclic (napthenic) alkanes [29]. The more aromatic fractions are called asphaltenes that are typically of a higher molar weight and contain fused ring systems that could be associated with small amounts of metals such as nickel, vanadium, and iron [29].

There is a significant amount of ambiguity in the literature as the asphaltenes fraction, defined by its insolubility in n-heptane, can also be contaminated with paraffin of high enough molar weight that makes it co-precipitate [30]. The general consensus is that the asphaltenes fraction together with the paraffin slowly precipitates out into a sol-type, sol/gel-type, or gel-type structure that, depending on temperature, viscosity, and composition, takes from days to weeks or months to equilibrate [16,17,18,19,31,32,33,34,35,36,37,38,39,40].

Blokker and Van Hoorn [33] coined the term “physical hardening” and stated that it involves the rather rapid crystallization of waxes and the slower precipitation of asphaltenes. Binders with high contents of both linear paraffins (wax) and asphaltenes are most susceptible to cracking distress as, due to their gelled state at ambient and low temperatures, they are unable to relax thermal and traffic-induced stresses [38,40,41] and suffer from weak spots at the somewhat sharp interface between the crystalline and amorphous phases [42,43,44].

Current specifications in most of Canada and the United States are based on the work done under the U.S. Strategic Highway Research Program (SHRP) [45]. The product of SHRP was the Superpave™ specification, which grades asphalt binders at high, intermediate, and low temperatures to control rutting, fatigue, and thermal cracking distress [45].

At high temperatures, Superpave sets a lower limit on the complex modulus divided by the sine of the phase angle, G*/sinδ, which for straight run binders is close to the complex viscosity [46]. It is generally accepted that the high temperature Superpave grade is reasonably effective at controlling rutting distress, although it should be recognized that aggregate structure and pavement thickness are two factors that can often be more important than binder properties.

At intermediate temperatures, an upper limit of 5 MPa is set on the loss modulus, G*sinδ, for a residue aged for 20 h in a pressure aging vessel (PAV) at a temperature of 100 °C and pressure of 2.08 MPa [45]. It has been found that the intermediate temperature Superpave grade is unable to correlate well with fatigue performance largely because it confounds the beneficial effects of viscous energy dissipation and the formation of damage [47,48,49,50]. It also favors the use of binders with low phase angle and low stiffness, which are known to suffer more from oxidative age hardening, phase separation, and exudative aging [51].

At low temperatures, the Superpave specification sets limits on the creep stiffness, measured in the BBR in three-point bending at 60 s of loading, S(60 s), and creep rate (i.e., the slope of the logarithmic creep stiffness master curve) also measured after 60 s of loading, m(60). The maximum S(60 s) of 300 MPa came from a decision to increase it from 200 MPa, as otherwise, too many asphalt binders sold at the time of SHRP would not have met the specification [45]. The original 200 MPa limit was based on a single field validation study many years earlier by Readshaw [52] in British Columbia. The m-value limit of 0.300 was a late addition to the specification that was based on the average for the relaxation rate at the limiting stiffness temperature for the eight core asphalt binders used in SHRP. The m(60 s) value was intended to prevent the use of heavily air-blown binders that were known to suffer from reduced creep, reduced stress relaxation, and exudative aging.

In general, asphalt binders are highly susceptible to changes in temperature and, due to their high viscosity at and below room temperature, are often graded in a state of non-equilibrium. This problem is one that has confounded pavement design since the work of Dow [14,15], Hubbard and Pritchard [16], Traxler [17,18,19], and others [53,54,55,56,57,58,59,60,61,62], and was similarly of concern to SHRP researchers developing the BBR [35,36]. Hence, the SHRP program spent a considerable amount of time and resources investigating physical hardening phenomena (thermoreversible aging). An early draft of the Superpave specification contained an option to test binders after one and 24 h of conditioning at the test temperature, but for reasons that are not well documented that provision never found wide acceptance [45].

Shortly after the complete implementation of the Superpave binder specification in Ontario, the Ministry of Transportation of Ontario (MTO) initiated research projects to investigate resultant widespread premature cracking around eastern and northeastern parts of the province [3,4,5,6]. These investigations eventually resulted in an improved asphalt cement specification by incorporating both the DENT and EBBR tests [63,64].

The DENT test provides an approximate critical crack tip opening displacement (CTOD), which is a value for the strain tolerance of binders in their ductile state and is highly correlated with fatigue cracking performance [50]. The CTOD is best described as a somewhat improved measurement of ductility. It is measured under more severe constraint in deeply notched specimens and at lower temperatures compared to a regular ductility test. However, it should be noted that there is a high correlation between CTOD and conventional ductility [12,65]. The DENT test provides essential and plastic works of failure in addition to the CTOD and the relevance of those to pavement performance remains to be better understood.

The EBBR was developed to be as similar as possible to the regular BBR test. It conditions samples for 1, 24, and 72 h at T_d_ + 10 and T_d_ + 20, where T_d_ is the design temperature of the pavement according to climatic requirements [2,3,4,5]. The 72 h grade loss from the one-hour result at T_d_ + 10 (roughly equal to the AASHTO M320 grade) is calculated and serves as a measure of durability. Grade losses found for tank and recovered binders range approximately from 0 to 15 °C, depending on the quality and durability of the binder [5,6,7].

While both the DENT and EBBR have proven to provide much enhanced performance grading, there is a need to find simpler test methods that can be completed in less time with less material to replace these protocols for future specifications. To that end, a butt joint test (BJT) for the ductile performance grading of asphalt binders was recently proposed [13]. Introducing the BJT as an alternative method for the DENT was able to significantly reduce material requirement and testing time. Compared with the DENT test, the BJT only takes 30 min and at the same time provides precise, sensitive, and accurate ductile strain tolerances. However, the BJT test has a problem with stiff binders that can generate tensile loads that exceed the capacity of the rheometer [13]. Hence, in this research a tertiary tensile creep test is investigated, which provides a measure of strain tolerance within the load capacity of the rheometer used. In addition, dynamic and creep shear tests are conducted at ambient and just below ambient temperatures in order to provide a comparison with results obtained in the EBBR protocol at much lower temperatures.

In spite of the numerous investigations that have come to similar conclusions to those found in the seminal publications by Traxler and coworkers [17,18,19] on thermoreversible aging, the problems created by this gradual change in properties in specification grading remain to be effectively sorted out today. This paper focusses on the measurement of phase angle as it has been shown in previous studies to be highly correlated to EBBR limiting grades and field cracking performance [66,67,68,69,70,71]. Testing of the extracted and recovered asphalt binder allows for the proper acceptance of materials as placed in the contract, thus facilitating the proper and responsible design for a true circular economy.

## 3. Experimental

### 3.1. Materials

A total of 32 asphalt binders were used in this study from hot mix asphalt (HMA) provided by five different user agencies. Samples were shipped directly by the user agency to Queen’s University by overnight courier and processed within a maximum of 1–2 months.

### 3.2. Methods

Binders were extracted using dichloromethylene (DCM) solvent [8,9]. All solutions were twice fed through a high-speed centrifuge (Ploog Engineering, Crown Point, IN, USA) to remove the fines. Recovery of the asphalt binder was done under a dry nitrogen gas atmosphere at moderate to high vacuum in a rotary evaporator (BUCHI Corporation, New Castle, DE, USA). Once no further DCM was visibly being distilled, the temperature of the oil bath was raised to 160 °C and the flask was subjected to vacuum below 50 mbar for an additional one hour. Asphalt binders were aged in a pressure aging vessel (PAV, Prentex, Dallas, TX, USA) according to standard procedures embodied in AASHTO R 28–09 [72] before testing at intermediate and low temperatures.

Small amounts of PAV residues were mounted in the dynamic shear rheometer (DHR-1 or DHR-2, TA Instruments, New Castle, DE, USA) at 64 °C and rapidly brought to 34 °C for testing at a film thickness of 2 mm. Samples were equilibrated for 10 min at each test temperature prior to testing at 12 °C intervals from 34 °C to −2 °C to determine the intermediate temperature complex modulus, G*, and phase angle, δ. Test frequency and strain level were kept constant at 10 rad/s and 0.1%, respectively.

The PAV residues were tested according to the DENT protocol described in AASHTO method TP 113-15 [73]. In brief, samples were poured in silicone molds with aluminum end inserts to facilitate shear transfer of the load from the test frame to the specimen. Notch depths varied to provide ligaments of 5, 10, and 15 mm in 10 mm thick specimens. The total works of failure were divided by the ligament area and plotted versus ligament length. The extrapolated intercept provided the essential work of failure, which was subsequently divided by the net section stress in the smallest ligament to determine the CTOD.

The PAV residues were tested in the EBBR protocol as described in AASTHO method TP 122-16 [74]. In brief, six samples each were conditioned at T_d_ +10 and T_d_ + 20 (where T_d_ is the design temperature of the pavement), for 1, 24, and 72 h. After each conditioning time, the samples were tested at T_d_ + 10 and T_d_ + 16 to determine pass and fail properties. From the individual stiffness and m-value measurements, actual grade temperatures were calculated by interpolation or extrapolation. The limiting low temperature grade (LLTG) was determined as the warmest of all limiting grade temperatures and the grade loss (GL) was determined as the difference between the 1 h limiting grade at T_d_ + 10 and the LLTG [74].

All PAV residues were also tested in the dynamic hybrid rheometer as 8 mm diameter by 0.5 mm thin films according to the three-in-one protocol. First, samples were mounted at 64 °C and after equilibration for 10 min at 35 °C, tested at 10 °C intervals from 35 °C to −5 °C, at frequencies of 0.1, 0.316, 1, 3.16, and 10 rad/s at a strain level of 0.1%. From these results, the temperature at which the phase angle at 10 rad/s reached 30° was calculated as a low temperature performance grade. Second, the same samples were subsequently equilibrated at 0 °C and tested for 240 s in creep shear at 1000 Pa followed by recovery for 760 s. Next, the creep test was repeated at 5 °C. From the two creep tests in shear, the temperature at which the creep rate, m, reached 0.5, was calculated according to the following equations [75]:

(1) $$\log\;S^{\prime }(t)=A+B\left[\log(t)\right]+C{\left[\log(t)\right]}^{2}$$

(2) |m|=B+2C[log(t)]

where S′(t) is the time-dependent shear creep stiffness, m is the creep rate in shear, t is the time in seconds, A, B, and C are regression coefficients.

**Figure 2:**
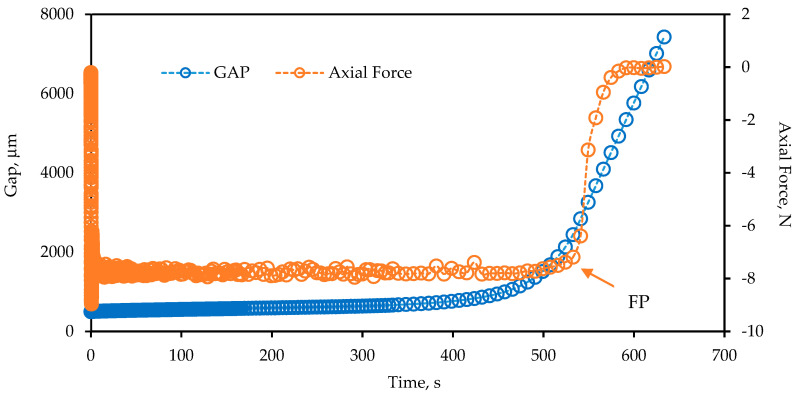
Tertiary creep test results on a 0.5 mm film to measure the failure point (FP).

Finally, the same samples as measured in the first two steps of the three-in-one protocol were subsequently equilibrated at 15 °C and subjected to a tensile creep load of 8 N to failure in order to determine strain tolerance in the ductile state. Figure 2 provides a representative tertiary creep test result with an illustration of how the failure point was determined at the sudden loss of the creep load control.

The results from the three-in-one protocol to determine phase angle, creep rate and ductile failure strain were compared with findings from the standard DSR, DENT and EBBR tests. The advantage of the three-in-one protocol is obvious as it uses less than a gram of material versus approximately 150 g for the combined DENT/EBBR testing, it is automated and might therefore be more repeatable, and produces similar insights as the DENT/EBBR.

## 4. Results and Discussion

### 4.1. Oscillatory Shear Testing

**Figure 3:**
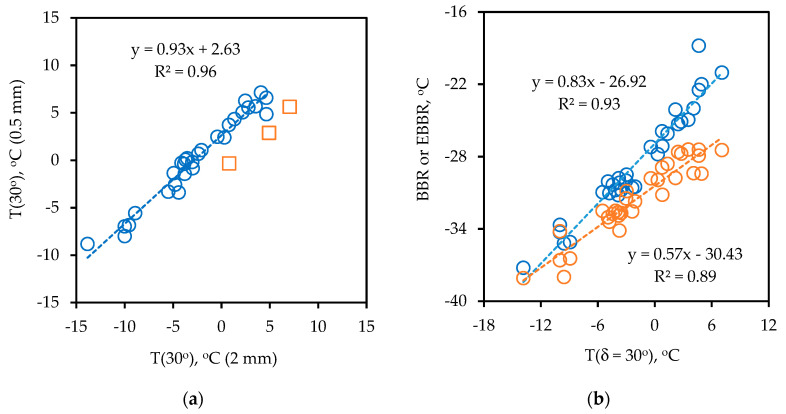
(**a**) Reproducibility of limiting phase angle temperature determinations using 2 and 0.5 mm thin films in the DSR; (**b**) Correlation between limiting phase angle temperature T(δ = 30°) (2 mm) and BBR (red symbols) and EBBR (blue symbols) limiting grades.

The limiting phase angle temperatures determined as part of the intermediate temperature Superpave grading and the three-in-one protocol are given in Figure 3a. It is obvious that there is a strong correlation and that the reproducibility is high (short of three outliers in squares). Tests were done by the same person several months apart. The results for the two film thicknesses are 2.63 °C apart, which is due to the thinner film being more constrained and thus less able to flow.

The correlation between the limiting phase angle temperature and the BBR and EBRR grades are in Figure 3b. These comparisons show that there are strong similarities, but that the T(δ = 30°) and EBBR have a higher sensitivity (wider range) compared to the regular BBR. The slope for the EBBR straight line fit is about 46% higher than what it is for the regular BBR (0.83 versus 0.57), which reflects the significantly improved sensitivity for the T(δ = 30°) and EBBR. The EBRR LLTG and T(δ = 30°) are most closely correlated, suggesting that cracking control may be achieved with similar efficiency when utilizing a T(δ = 30°) acceptance criterion. The difference for this limited set of data is in agreement with data from previous studies on other binders [8,11,76,77]. However, a switch from the somewhat lengthy EBBR protocol to the more practical T(δ = 30°) would mean that the information provided by the grade loss is lost. It has also been recognized that for more severely aged material, the limiting phase angle temperatures also start to suffer from the effects of thermoreversible aging [78,79].

### 4.2. Creep Testing

**Figure 4:**
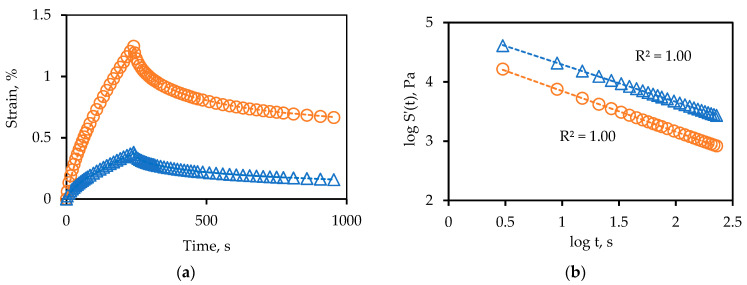
(**a**) Raw and (**b**) processed shear creep test results at 1000 Pa and two temperatures.

**Figure 5:**
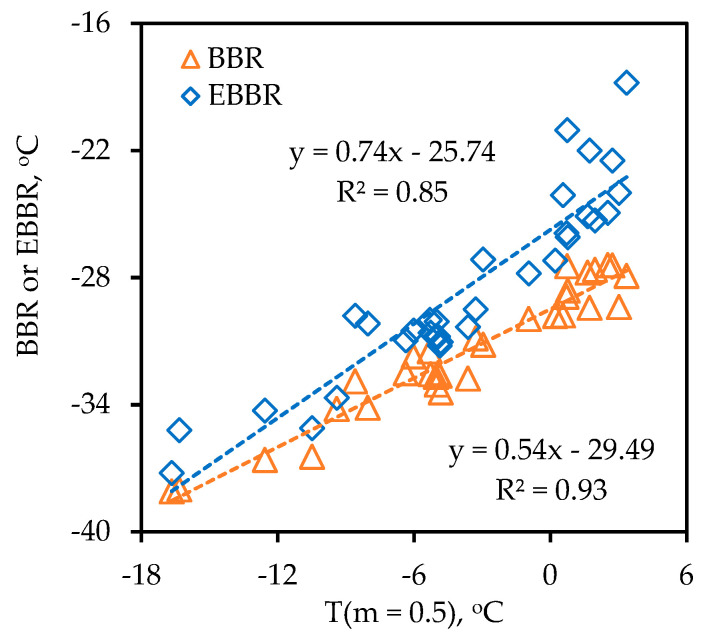
Correlation between T(m = 0.5) and limiting BBR and EBBR temperatures.

Representative creep test results for one of the binders are in Figure 4. Use of Equation (1) provides a high correlation with the raw displacement data. The correlation between T(m = 0.5) and BBR and EBBR is provided in Figure 5. As can be seen, the creep data fit Equation (1) with a high degree of accuracy. The correlation between the limiting creep rate temperature, T(m = 0.5), and the BBR and EBBR limiting temperatures is also reasonably good. As for the phase angle data in Figure 3, the range for the T(m = 0.5) at 19.6 °C is significantly wider than what it is for the BBR at 10.7 °C, somewhat wider than what it is for the EBBR at 16.5 °C, but not quite as wide as the span for the T(δ = 30°) at 20.9 °C. A wider range with equal or better precision is beneficial in a grading protocol as it allows for the better differentiation between samples.

### 4.3. Tertiary Creep Testing

**Figure 6:**
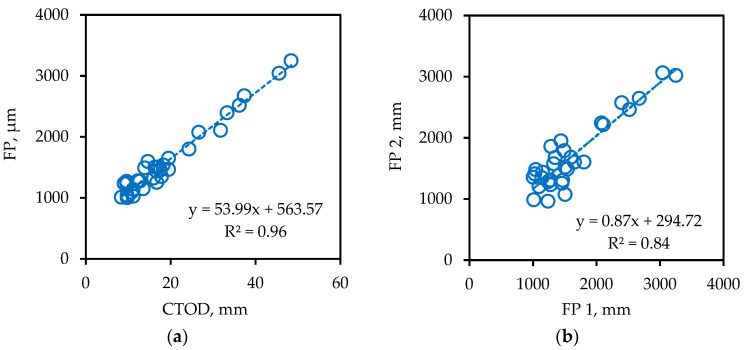
(**a**) Correlation between failure point (FP) and DENT CTOD; (**b**) Repeatability of the tertiary creep test (failure point FP2 is a repeat of FP1 as determined by one of the authors (Y.L.), at a time several months after the determination of FP1).

The final comparison is between the failure point in tertiary creep and the DENT CTOD as given in Figure 6a. The graph shows that there is a very high correlation and that both measurements provide nearly the same ranking. Figure 6b shows the repeatability for the tertiary creep test, which is also reasonable, although not as good as for the phase angles in Figure 3a.

## 5. Summary and Conclusions

Given the results and discussion presented, the following summary and conclusions are provided:

1. Constraint increases in thinner films and the limiting phase angle temperatures increase accordingly. However, there is a strong correlation between limiting temperatures measured in films of 0.5 mm (new protocol) and 2.0 mm (AASHTO M 320 standard) thickness.
2. The limiting phase angle temperature shows a very strong correlation with the EBBR LLTG temperature (R^2^ = 0.93), and a somewhat lesser correlation with the regular BBR temperature (R^2^ = 0.89).
3. The ranges for limiting T(δ = 30°) (20.9 °C) and EBBR (16.5 °C) temperatures for this set of 32 binders were about 91 and 46% improved over the range of the regular BBR temperature (10.7 °C). Hence, the limiting phase angle temperature is significantly more responsive to changes in binder properties than both the BBR and EBBR.
4. The phase angle reflects the binder’s ability to relax thermal and traffic induced stresses and will therefore provide a good correlation with pavement cracking performance. Those binders that are of a gel type (low phase angle) are expected to perform poorly in service, while those binders that are of a sol type (high phase angle) are expected to perform well.
5. If and how a measure of binder stiffness needs to be included in the specification needs careful deliberation and further investigation through field monitoring of the investigated materials.
6. The DENT CTOD can be approximated with a high degree of accuracy by the failure point in the tertiary creep test. Whether and how this property needs to be included in future cracking specifications deserves further investigation through careful study of the long-term performance of the investigated materials.

Given the pervasiveness and seriousness of premature and excessive pavement cracking in cold climates, it is up to the user agencies to make the best use of the information provided.

## Data Availability

The raw/processed data required to reproduce these findings cannot be shared.
